# Loss of *p53 Ser18* and *Atm* Results in Embryonic Lethality without Cooperation in Tumorigenesis

**DOI:** 10.1371/journal.pone.0024813

**Published:** 2011-09-27

**Authors:** Heather L. Armata, Punita Shroff, David E. Garlick, Krista Penta, Andrew R. Tapper, Hayla K. Sluss

**Affiliations:** 1 Division of Endocrinology, Department of Medicine, University of Massachusetts Medical School, Worcester, Massachusetts, United States of America; 2 Department of Cancer Biology, University of Massachusetts Medical School, Worcester, Massachusetts, United States of America; 3 Brudnick Neuropsychiatric Research Institute, University of Massachusetts Medical School, Worcester, Massachusetts, United States of America; University of Michigan School of Medicine, United States of America

## Abstract

Phosphorylation at murine Serine 18 (human Serine 15) is a critical regulatory process for the tumor suppressor function of p53. p53Ser18 residue is a substrate for *ataxia-telangiectasia mutated* (ATM) and *ATM-related* (ATR) protein kinases. Studies of mice with a germ-line mutation that replaces Ser18 with Ala (*p53^S18A^* mice) have demonstrated that loss of phosphorylation of p53Ser18 leads to the development of tumors, including lymphomas, fibrosarcomas, leukemia and leiomyosarcomas. The predominant lymphoma is B-cell lymphoma, which is in contrast to the lymphomas observed in *Atm^−/−^* animals. This observation and the fact that multiple kinases phosphorylate p53Ser18 suggest Atm-independent tumor suppressive functions of p53Ser18. Therefore, in order to examine p53Ser18 function in relationship to ATM, we analyzed the lifespan and tumorigenesis of mice with combined mutations in p53Ser18 and *Atm*. Surprisingly, we observed no cooperation in survival and tumorigenesis in compound *p53^S18A^* and *Atm^−/−^* animals. However, we observed embryonic lethality in the compound mutant animals. In addition, the homozygous p53Ser18 mutant allele impacted the weight of *Atm^−/−^* animals. These studies examine the genetic interaction of p53Ser18 and *Atm in vivo*. Furthermore, these studies demonstrate a role of p53Ser18 in regulating embryonic survival and motor coordination.

## Introduction

Ataxia telangiectasia (A-T) is a recessive childhood disease with no cure. Patients with ataxia telangiectasia exhibit pleiotropic symptoms, including ataxia, telangiectasia, early aging, radiosensitivity and susceptibility to cancer [1]. The predominant childhood malignancy is lymphoid malignancies, such a B-cell non-Hodgkin lymphoma and T-cell lymphoid tumors (T-cell lymphoma and T-cell acute lymphoblastic leukemia) [2]. Mutations in *ataxia-telangiectasia mutated* (*ATM*, the gene mutated in A-T) have also been detected in sporadic lymphoid tumors, such as T-cell prolymphocytic leukemia [3], [4], [5], B-cell chronic lymphocytic leukemia [6], [7], [8] and mantle cell lymphoma [9]. In addition, other tumors, including brain tumors and certain carcinomas, are also seen in patients with A-T [10]. Studies also indicate a role for malignancy in heterozygous carriers. For example, women who are heterozygous carriers for A-T are at an increased risk for breast cancer [11].

*Atm-null* mice have been generated and can phenocopy several aspects of the A-T disease. The *Atm-null* animals develop tumors, predominantly lymphomas [12], [13], [14], [15]. The tumor cell type that develops is mainly immature T-cell thymic lymphoblastic lymphoma [13], [15]. *Atm*-deficient animals have been shown to exhibit gross motor skill impairment [15]. In addition, cells from *Atm*-deficient mice can exhibit some of the neurodegeneration observed in A-T [16], [17]. Additional studies have demonstrated that *Atm*-deficient animals undergo early aging when crossed into a telomerase deficient background [15], [18]. Recently, a knock-in mouse has been generated corresponding to the common human *ATM^763del6^* observed in A-T patients [19]. The *ATM^763del6^* mutation produces an almost full-length protein that lacks kinase activity. These mice had a longer life-span than *Atm-null* mice as well as a reduction in the number of lymphomas. This observation points to the fact that the outcome of the disease depends on the nature of the *ATM* mutation in the patients [1].

The tumor suppressor function of ATM has been linked to its role in DNA repair and checkpoint function [20], [21], [22]. The checkpoint response is coupled in part to the phosphorylation of downstream effector molecules, such as the tumor suppressor p53 [23]. ATM activates p53 directly or indirectly through activation of its downstream kinase chk2, leading to p53-dependent responses such as transient T-cell cycle arrest, senescence or apoptosis.

p53 is a critical tumor suppressor mutated in over 50% of human malignancies. Regulation of p53 can occur through phosphorylation of the amino-terminal transactivation domain [24]. An important site for regulation of p53 function is the Ser15 (murine Ser18) residue, a substrate for ATM and ATR (*ATM-related*) protein kinases. Studies of mice with a germ-line mutation that replaces Ser18 with Ala (*p53^S18A^* mice) have demonstrated that phosphorylation of p53Ser18 is required for normal DNA damage-induced PUMA expression and apoptosis, but not for DNA damage-induced cell cycle arrest [25]. *p53^S18A^* mice developed lymphomas mostly of B-cell origin, which is in contrast to the T-cell lymphomas which develop in *Atm-null* mice. These mice also developed several malignancies, including fibrosarcoma, leukemia, leiomyosarcoma, and myxosarcoma, which are unusual in *p53-null* and *Atm-null* mice.

Thus, the phosphorylation site Ser18 on p53 contributes to tumor suppression and regulation of lifespan *in vivo*. The p53Ser18 moiety can be phosphorylated by additional kinases other than ATM, suggesting there may be ATM-independent roles for p53Ser18. Further support for this hypothesis is that p53Ser18 deficient animals (*p53^S18A^* mice) develop mostly B-cell tumors [26], which are not observed in *Atm^−/−^* mice. In addition, it has also been shown that p53 can have a tumor suppressive role in *Atm*-deficiency [26].

In order to examine the function of p53Ser18 in the process of tumor suppression independent of ATM, we generated and characterized compound *p53^S18A^* and *Atm*-deficient animals. Surprisingly, the status of p53Ser18 did not alter the survival of *Atm-null* or *Atm^−/+^* mice. The tumor onset or profile of *Atm-null* mice was also not affected by p53Ser18 status. However, we observed embryonic lethality in the compound mutant animals. Furthermore, cell cycle was greatly affected in cells from these animals. Interestingly, we observed a decrease in weight in compound *Atm* ^−/−^; *p53^S18A/S18A^* animals compared to *Atm* ^−/−^ mice. We present here our findings of the contribution of p53Ser18 to ATM-mediated tumor suppression. Furthermore, these studies confirm the importance of p53Ser18 in regulating tumorigenesis *in vivo*.

## Materials and Methods

### Ethics statement

All mice were housed in a pathogen-free facility accredited by the American Association for Laboratory Animal Care (AALAC). Mice were under protocol number 1032. Mice were bred under standard conditions with a 12-hour light/dark cycle, and were fed ad libitum with standard chow (Iso-Pro3000, Prolab). The Institutional Animal Care and Use Committee of the University of Massachusetts approved all studies using animals.

### Mouse strains and tumor analysis

The generation and genotyping of the *p53^S18A^* mice [25], *p53^−/−^* mice [27], *Atm^−/+^* and *Atm^−/−^* mice [15] have been previously described. Because *Atm^−/−^* mice are sterile, *Atm^−/−^*; *p53^S18A/+^* mice were interbred to obtain the genotype *Atm^−/−^*; *p53^S18A/S18A^*. Similar age-matched cohorts of *Atm^−/+^*, *Atm^−/−^*, *Atm^−/−^*; *p53^S18A/S18A^*, *Atm^−/−^*; *p53^S18A/+^*, *Atm^−/+^*; *p53^S18A/S18A^*, and *Atm^−/+^*; *p53^S18A/+^* mice were established. All mice were on a mixed 129SvEv/C57Bl6 background. Litters with greater than 5 animals were included in the offspring analysis. The survival and tumor data in the control (wild-type) mice has been published [26]. The mice in the survival analysis were observed twice a week for any signs of tumors or distress. Mice were sacrificed when a tumor was apparent or when the mice became unhealthy (severe weight loss, severe dermatitis, or pronounced lordosis). Some animals were included in the survival but not the tumor analysis because of post-mortem autolysis. The mice were examined by necropsy to detect tumors or other gross pathology and tissues were fixed in 10% formalin. Fixed tumors or organs were embedded in paraffin and sectioned. Sections were mounted on slides and stained with hematoxylin and eosin. Slides were examined by a board-certified veterinary pathologist.

### Cell culture and proliferation assays

Murine embryonic fibroblasts (MEFs) were generated from day 13.5 embryos. Since *Atm^−/−^* mice are sterile the compound mutant MEFs were obtained from an *Atm^−/+^*; *p53^S18A/S18A^* intercross. The MEFs were maintained in Dulbeccos' Modified Eagles Medium (DMEM) supplemented with 10% fetal bovine serum, 5 mM glutamine, and penicillin and streptomycin (Invitrogen). The MEFs were cultured at sub-confluence and were passaged no more than 4 times, unless otherwise indicated. Cellular proliferation/survival analysis was performed as described [25] with pass 2 MEFs. Briefly, 2×10^4^ MEFs were plated onto each well of a 6-well plate, and each day after plating MEFs from three plates of each genotype were fixed, stained with trypan blue and absorbance was determined. MEFs from two different intercrosses were used for the experiments. Experiments were performed in triplicate for each line and are presented as mean values with standard deviations.

### Rota Rod Experiment

The rotarod apparatus (Stoelting) was used to measure motor coordination and balance, as well as the ability of mice to improve motor skill performance with training. During training, mice were placed on a rotarod accelerating from 4–40 RPM over 5 minutes. Each mouse received ten trials (one trial every five minutes) and the latency to fall off the rotarod in each trial was measured. The following day, mice received three test trials on the accelerating rotarod with each trial separated by one minute.

### Data Analysis

Survival of mice was determined by Kaplan Meier analysis using JMP software (SAS, Cary, NC). Pair-wise comparison of the Kaplan Meier analysis was done using the Log Rank test. Statistical analysis on perinatal lethality was done comparing expected # per genotype for each litter compared to actual # per genotype for each litter using a student's t-test. MEF analysis was done using student's t-test on triplicate readings per time point. Average latencies to fall from the rotarod were compared using One-way Analysis of Variance followed by Tukey post-hoc tests. Statistical significance was set at *P*<0.05.

## Results

### Growth Properties of *Atm^−/−^*; *p53^S18A/S18A^* Cells and Animals

**Figure 1:**
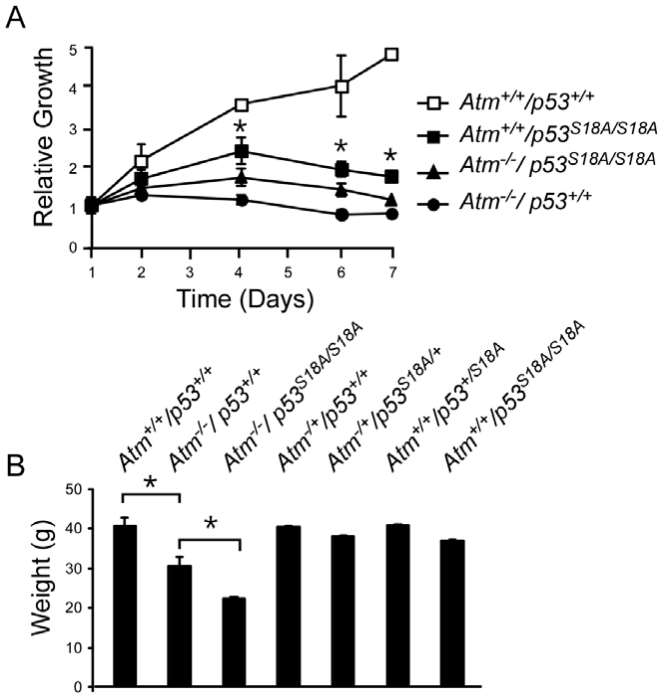
Analysis of growth and development in *Atm^−/−^*; *p53^S18A/S18A^* animals. **A.** *Atm^−/−^*; *p53^S18A/S18A^* MEFs exhibit a decreased proliferation. Growth comparison between wildtype, *Atm^−/−^* and *Atm^−/−^*; *p53^S18A/S18A^* MEFs (S18A = p53^S18A^, WT = wildtype). **B** *Atm^−/−^* mice exhibit reduced weight. Weight of animals compared at 5–6 months of wildtype (n = 10); *Atm^−/−^* (n = 5); *Atm^−/−^*; *p53^S18A/S18A^* (n = 8); *Atm^−/+^*; *p53^+/+^* (n = 12); *Atm^−/+^*; *p53^S18A/+^* (n = 10); *Atm^+/+^*; *p53^S1A8/+^* (n = 9); and *Atm^+/+^*; *p53^S18A/S18A^* (n = 12). The data presented are the mean ± S.E.M. Statistically significant differences are indicated with an asterisk (*, *P*<0.05). n = # of animals.

Cells from *Atm*-deficient animals exhibit decreased growth properties, characterized by decreased cell proliferation [12]. We compared the growth properties of *Atm^−/−^*, *p53^S18A/S18A^* and compound *Atm^−/−^*; *p53^S18A/S18A^* MEFs. Figure 1A demonstrates the results from a representative experiment, and cell lines were plated in triplicate. *Atm^−/−^* MEFs exhibited a decrease in trypan blue staining, which is correlative of slower proliferation, compared to wild-type cells, as previously reported [12], [15]. *p53^S18A/S18A^* MEFs also exhibited decreased growth compared to wild-type cells as previously described [25]. Cell lines generated from heterozygous mice (*p53^S18A/+^* and *Atm^−/+^*) grew similar to wildtype cells (data not shown). The *p53^S18A/S18A^* cells exhibited greater rate of growth than *Atm^−/−^* cells (**Figure 1A**). Importantly, the cells used were early pass MEFs which do not exhibit the significant number of senescenT-cells observed in higher pass cells. Interestingly, the compound MEFs exhibited a proliferation rate that was slower than *p53^S18A/S18A^* MEFs, but not as slow as *Atm^−/−^* MEFs (**Figure 1A**). This suggested there was no cooperation in the cell growth defects observed in the compound MEFs as they did not grow slower than single mutant (*Atm^−/−^*) MEFs. However, there was a decrease on the growth of *p53^S18A/S18A^* MEFs with the loss of *Atm*, suggesting an additional site of ATM regulation on the p53^S18A^ protein.

*Atm^−/−^* mice exhibit additional growth defects including decreased body weight [13], [14], [15]. We observed a significant reduction in weight in *Atm^−/−^* animals compared to wildtype animals at 5–6 months of age (**Figure 1B**). There was a further significant reduction in body weight of *Atm^−/−^*; *p53^S18A/S18A^* animals compared to *Atm^−/−^* animals. Importantly, *p53^S18A^* homozygous or heterozygous mice had a body weight similar to wildtype. Thus, the presence of the p53Ser18 mutation synergizes with loss of Atm in providing a growth advantage.

### Prenatal Lethality in *Atm^−/−^*; *p53^S18A/S18A^* Animals

It has been reported that loss of *p53* in an *Atm*-deficient background leads to embryonic lethality [28]. The frequency of *Atm^−/−^* offspring from 13 *Atm* ^−/+^ intercrosses was not significantly reduced (**Table 1**, Cross A), confirming that loss of Atm does not lead to embryonic lethality. Importantly, we previously observed no embryonic lethality due to the p53^S18A^ mutation [26]. To determine whether p53Ser18 loss would lead to perinatal lethality in an *Atm-null* background, *Atm^−/+^*; *p53^S18A/S18A^* mice were intercrossed and genotype of the offspring was analyzed (**Table 1**, Cross B). The number of *Atm^−/−^*; *p53^S18A/S18A^* offspring was significantly reduced in 25 litters, from the expected 25% to 16.9%. Thus, 30 out of the predicted 45.75 *Atm^−/−^*; *p53^S18A/S18A^* mice were born, indicating about 35% of *Atm^−/−^*; *p53^S18A/S18A^* offspring die prenatally. This percent is less than the 60% of *Atm^−/−^*; *p53^−/−^* mice which have been reported to die prenatally [28]. However, it has been reported up to 15% of *p53^−/−^* mice exhibit embryonic lethality [29]. Thus, although there is a prenatal lethality associated with *p53^S18A^*, it is not as robust as that with loss of *p53*.

**Table 1:** Genotype of Offspring from Intercrosses.

*Cross A: Atm^−/+^; p53^+/+^*×*Atm^−/+^*; *p53^+/+^* Total n = 202						
Genotype:	*Atm^+/+^* ; *p53^+/+^*		*Atm^−/+^* ; *p53^+/+^*		*Atm^−/−^* ; *p53^+/+^*	
Obtained	46	(22.8%)	116	(57.4%)	40	(19.8%)
Expected	50.5	(25%)	101	(50%)	50.5	(25%)
**T-Test**	**0.43**		**0.08**		**0.12**	

*Cross B: Atm^−/+^*; *p53^S18A/S18A^*×*Atm^−/+^*; *p53^S18A/S18A^* Total n = 183						
Genotype:	*Atm^+/+^* ; *p53^S18A/S18A^*		*Atm^−/+^* ; *p53^S18A/S18A^*		*Atm^−/−^* ; *p53^S18A/S18A^*	
Obtained	50	(27.32%)	103	(56.28%)	30	(16.39%)
Expected	45.75	(25%)	91.5	(50%)	45.75	(25%)
**T-Test**	**0.58**		**0.34**		**0.007**	

### Analysis of mouse survival

**Figure 2:**
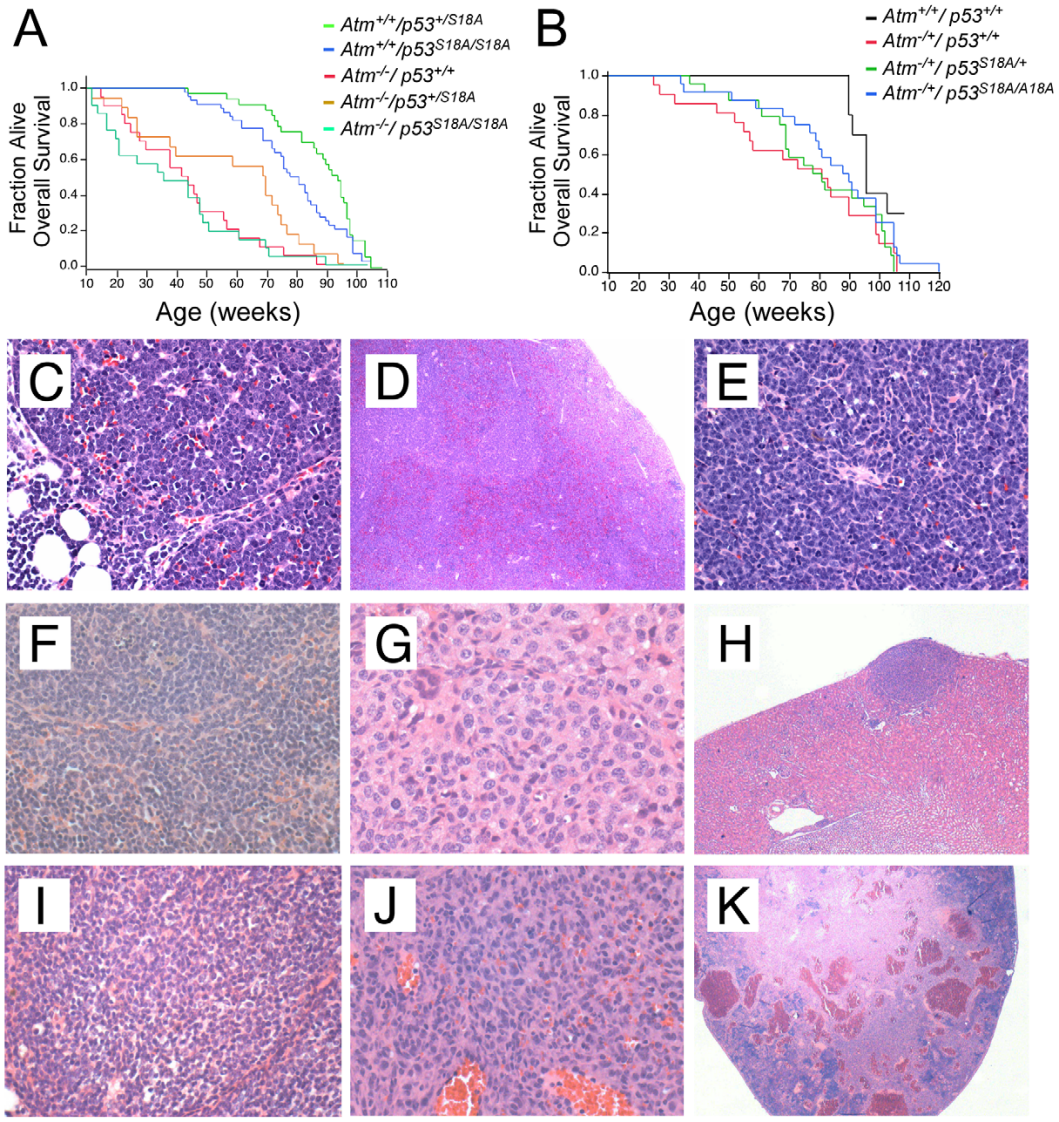
Survival and tumorigenesis of compound *Atm* deficient and *p53^S18A^* mice. (**A–B**) Kaplan-Meier distribution of overall survival of (**A**) *Atm^−/−^*; *p53^+/+^* (n = 21); *Atm^−/−^*; *p53^S18A/+^* (n = 19); *Atm^−/−^*; *p53^S18A/S18A^* (n = 22); *Atm^+/+^*; *p53^S18A/+^* (n = 31); and *Atm^+/+^*; *p53^S18A/S18A^* (n = 42) mice. (**B**) *Atm^+/+^*; *p53^+/+^* (n = 12), *Atm^−/+^*; *p53^+/+^* (n = 21), *Atm^−/+^*; *p53^S18A/+^* (n = 24), and *Atm^−/+^*; *p53^S18A/S18A^* (n = 24) mice over the course of two years. Percent survival is on the y-axis and age of death (in weeks) is on the x-axis. Death was by either presence of tumor, illness or case unknown. n = # of animals. (**C–E**) **Highly invasive lymphoma in an** ***Atm^−/−^*** **;** ***p53^S18A/S18A^*** **animal.** **C.** A lymphoblastic lymphoma comprised of monomorphic cells showing complete effacement of the thymus (20×) **D.** Spread of lymphoma to the spleen with effacement of splenic architecture (4×). **E.** Higher magnification of the lymphoma in spleen showing malignant lymphocytes (20×). (**F–H**) **Tumors in** ***Atm^−/+^*** **;** ***p53^S18A/+^*** **animals.** **F.** Marginal lymphoma in the spleen of one animal (40×). **G.** Abdominal histiocytic sarcoma in one animal showing some spindle shaped cells and characteristic macrophages/histiocyte cells (20×). **H.** Histiocytic sarcoma from same animal invading the kidney (4×). (**I–K**) **Tumors in** ***Atm^−/+^*** **;** ***p53^S18A/S18A^*** **animals.** **I.** Lymphoma present in the thymus of one animal, visible as a diffuse sheet of lymphocytes (20×). **J.** Hemangiosarcoma present in the spleen of another animal (4×). **K.** Higher magnification of hemangiosarcoma in spleen showing almost complete effacement of the spleen (20×).

To examine the role of p53Ser18 in an ATM-independent survival, we performed a survival analysis to examine the effects of *p53^S18A^* in the survival of *Atm^−/−^* and *Atm^−/+^* mice. The *Atm^−/−^* mice had a rapid demise compared to wildtype mice (**Figure 2A, B**). Of note, we observed a median survival of the *Atm^−/−^* mice of 41 weeks, which has been reported for the mice in the same genetic background and mouse facility conditions [30]. Surprisingly, Kaplan Meier analysis indicated that the presence of the homozygous *p53^S18A^* mutation had no significant effect on the survival of *Atm^−/−^* mice (**Figure 2A**). However there was a reduction in the survival of *Atm^−/−^*; *p53^S18A/S18A^* mice compared to *p53^S18A/S18A^* mice [26]. The presence of a heterozygous p53^S18A^ mutation also had no effect on the overall survival of *Atm^−/−^* mice, although the median was delayed (but not significantly) (**Figure 2A**). The lack of cooperation in tumorigenesis is in contrast to that observed for *p53^−/−^* animals, which demonstrate cooperation in survival and tumorigenesis with *Atm^−/−^* mice [28].

We observed a significant decrease in the overall survival of *Atm^−/+^* mice compared to wildtype mice (**Figure 2B**). In addition, there was a significant decrease in the median survival. The wildtype median survival age was 98 weeks, whereas the *Atm^−/+^* median survival age was 81 weeks. The mutation of one or two alleles of p53Ser18 had no significant effect on the overall survival of *Atm^−/+^* mice (**Figure 2B**). The median survival age for *Atm^−/+^*; *p53^S18A/S18A^* and *Atm^−/+^*; *p53^S18A/+^* mice was 90 and 81 weeks respectively. Interestingly, we previously observed the median survival age for *p53^S18A/+^* mice was 94 weeks [26], thus, the loss of one allele of *Atm* further decreased lifespan of *p53^S18A/+^* mice. This suggested that the residual ATM in *Atm^−/+^*; *p53^S18A/+^* mice could be phosphorylating the remaining p53Ser18 moiety, which would explain the increased viability of *p53^S18A/+^* animals compared to *p53^S18A/S18A^* animals [26].

### Analysis of Spontaneous Tumorigenesis in *Atm* and p53Ser18 Deficient Mice

We analyzed spontaneous tumor development in *Atm^−/−^* and *Atm^−/−^*; *p53^S18A/S18A^* animals. The predominant tumor observed in these animals was lymphoma (**Table 2**). Among all tumors, the incidence of lymphomas was 75% in *Atm^−/−^* mice, 100% in *Atm^−/−^*; *p53^S18A/S18A^* mice and 91% in *Atm^−/−^*; *p53^S18A/+^* mice. The age of onset was similar for all genotypes. Lymphoma was detected in the thymus and the surrounding organs and tissues in all genotypes, reflecting the aggressive nature of tumors found in *Atm^−/−^* mice [15]. By routine histology, there was a similar morphological appearance in tumors of the three genotypes. In *Atm^−/−^* animals, the majority of thymic lymphomas had a lymphoblastic appearance, which, along with the thymic involvement, was consistent with T-cell origin. Organs that were affected as well also exhibited a lymphoblastic morphology. In addition, poorly differentiated lymphomas were also observed in some thymi in *Atm-null* animals. In these animals the lymphomas in the surrounding tissue also had a more poorly differentiated appearance. The majority of lymphomas in the thymus in *Atm^−/−^*; *p53^S18A/S18A^* mice were also lymphoblastic and any invaded surrounding tissue also had a lymphoblastic profile. Representative micrographs of a highly infiltrative lymphoma in an *Atm^−/−^*; *p53^S18A/S18A^* animal are shown in **Figure 2C–E**.

**Table 2:** Tumorigenesis in *Atm* and *p53Ser18* deficient animals.

	*Atm^−/−^* *p53^+/+^*	*Atm^−/−^* *p53^S18A/+^*	*Atm^−/−^* *p53^S18A/S18A^*	*Atm^−/+^* *p53^+/+^*	*Atm^−/+^* *p53^+/S18A^*	*Atm^−/+^* *p53^S18A/S18A^*
Mice Analyzed	11	16	16	15	14	18
Mice with tumors	8	12	12	3	5	3
Total tumors	8	13	13	3	5	4
Tumor Classification						
Lymphoma	6	12	12	2	3	1
Histiocytoma	0	1	0	0	0	0
Histiocytic sarcoma	0	0	0	0	1	0
Hemangiosarcoma	0	0	0	1	0	1
Hemangioma	0	0	0	0	1	0
Fibrosarcoma	0	0	0	0	0	1
Carcinoma	1	0	0	0	0	0
Adenoma	1	0	0	0	0	1

Mice on the *Atm^−/−^* background developed few cancers other than lymphoma (**Table 2**). One bronchoalviolar carcinoma was detected in an *Atm^−/−^* animal, and this animal also had a lymphoblastic lymphoma in the thymus. In addition, one malignant histiocytoma was observed in an *Atm^−/−^*; *p53^S18A/+^* animal.

We observed a shortened life-span of *Atm^−/+^* mice compared to wildtype animals (**Figure 2B**). We determined if *Atm^−/+^* and compound *Atm^−/+^*; *p53^S18A^* animals also developed spontaneous tumors. We detected an increase of tumor incidence in *Atm^−/+^* animals compared to wildtype animals. Whereas 23% of *Atm^−/+^* animals developed tumors, only 8% of wildtype mice developed malignant tumors [26]. The tumors detected in *Atm^−/+^* mice were lymphomas and a hemangiosarcoma (**Table 2**). A follicular lymphoma in one animal involved the spleen, liver and also mandibular lymph node. A poorly differentiated lymphoma in another animal involved the liver and the spleen. The observed hemangiosarcoma involved the spleen of one animal and had ruptured.

The *Atm^−/+^*; *p53^S18A/+^* animals also developed tumors, with a penetrance of 35%. One animal had a marginal zone lymphoma in the spleen (**Figure 2F**). Another two animals had follicular cell lymphomas. One animal had a hemangioma in the liver. In addition, one animal had an abdominal mass that was a histiocytic sarcoma (**Figure 2G**) that had spread to the kidney (**Figure 2H**).

The tumor penetrance for *Atm^−/+^*; *p53^S18A/S18A^* animals was 16%. Interestingly, very few lymphomas were observed in *Atm^−/+^*; *p53^S18A/S18A^* mice. This observation was in contrast with what we had observed in *p53^S18A/S18A^* animals where 35.7% of animals had tumors, and 81% of tumors were lymphomas [26]. There was one incipient lymphoma present in the thymus of one *Atm^−/+^*; *p53^S18A/S18A^* animal, visible as a diffuse sheet of neoplastic lymphocytes (**Figure 2I**). In addition, other animals developed an adenoma, a fibrosarcoma, and a hemangiosarcoma in the spleen. The hemangiosarcoma almost completely effaced the spleen (**Figure 2 J,K**).

We previously observed tumor-free *p53^S18A^* animals that presented with cellular alterations at the time of death that were consistent with accelerated lifespan [26]. We observed some of the same alterations in cellular composition in the compound *Atm^−/+^*; *p53^S18A^* animals [26]. In the *Atm^−/+^*; *p53^S18A/+^* mice we observed alterations in the kidney, such as increased inflammation, tubular cysts, hematomas and fibrosis. The *Atm^−/+^* mice also presented with multifocal inflammation in liver. The *Atm^−/+^*; *p53^S18A/S18A^* mice had liver inflammation and degeneration. Kidneys also had increased inflammation as well as glomeruloneropathy. Further analysis of a larger cohort of animals will be required to determine if the animals are presenting with accelerated aging, as we observed in the *p53^S18A^* animals [26].

### Performance on the rota rod apparatus

**Figure 3:**
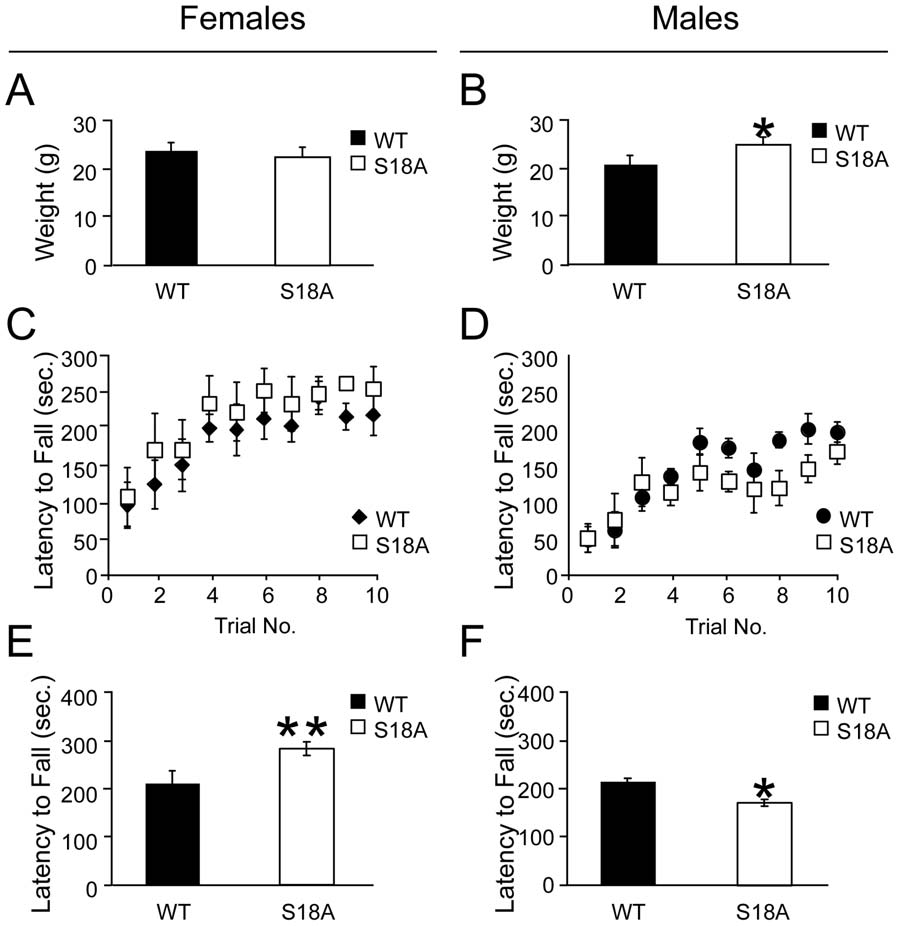
Performance on an accelerating rotarod. Performance of wildtype (WT) and *p53^S18A/S18A^* animals on a rotarod apparatus. Data for female mice is on the left and male mice on the right. **A–B** Weight of animals tested. Data are presented as mean ± standard deviation. **C–D** Training of animals on rotarod. 4–5 month old animals were trained for 10 trials separated by 5 min. on an accelerating rotarod (0–40 rpm). The latency to fall was recorded (n = 5–6 mice/genotype/gender). **E–F** Average of three experimental trials 24 hrs. after training. Data are presented as mean ± standard deviation. * *P*<0.05, ** *P*<0.01. Data were analyzed by two-tailed student's t-test (A, B) and One-Way ANOVA followed by Tukey post-hoc (C–F).

ATM has been shown to contribute to motor coordination [15]. We therefore tested motor coordination in *p53^S18A^* animals to determine if Ser18 contributes to this function. The accelerating rotarod assay was used to measure motor coordination in *p53^S18A^* mice. All data were collected on age- and gender-matched naïve animals. Immediately prior to training, mice were weighed. Female wildtype and *p53^S18A^* mice did not significantly differ in weight; whereas male *p53^S18A^* mice were modestly but significantly heavier than wildtype mice (**Figure 3A, B**). During the training phase, mice were placed on the accelerating rotarod for ten consecutive trials over the course of one hour and latency to fall off of the rotarod was recorded. Latency to fall off of the rotarod in both wildtype and *p53^S18A^* mice increased with successive trials and reached a plateau by the tenth trial (**Figure 3C, D**). Twenty-four hours after training, mice were again placed on the accelerating rotarod and their performance was measured. Compared to wildtype female animals, female *p53^S18A^* animals exhibited an increased latency to fall off of the rotarod (**Figure 3E**, F_(1, 9)_ = 31.6, p<0.001). Conversely, male *p53^S18A^* mice exhibited a significant decreased latency to fall off of the rotarod compared to male wildtype mice (**Figure 3F**, F_(1,9)_ = 5.8, p<0.05).

## Discussion

ATM has been shown to have a role in DNA repair, DNA-damage induced checkpoints, and telomere maintenance. p53 is a substrate of ATM that is intimately linked with its role in DNA repair and checkpoint function. We previously generated mice with a germ-line mutation that replaces Ser18 with Ala (*p53^S18A^* mice). Studies with these mice and the same mice generated by another lab have demonstrated that phosphorylation of p53Ser18 is required for normal DNA damage-induced PUMA expression and apoptosis, but not for DNA damage-induced cell cycle arrest [25], [26], [31]. In addition, *p53^S18A^* mice developed late-onset lymphomas at 18 months of age [26]. Studies indicate an ATM-independent tumor-suppression function of p53 [32]. Our observation that *p53^S18A^* mice develop predominantly B-cell tumors whereas *Atm^−/−^* mice develop T-cell tumors lead us to hypothesize that there is an ATM-independent growth suppressive role for p53Ser18. We investigated the role of p53Ser18 in the tumor suppressor function of ATM by generating and characterizing compound mutant animals.

Cells generated from *Atm^−/−^* and *Atm^−/−^*; *p53^S18A/S18A^* animals grew slower than wildtype cells. In addition, *p53^S18A/S18A^* cells grew significantly faster than *Atm^−/−^* cells. However, there was no synergy in the *Atm^−/−^*; *p53^S18A/S18A^* cells compared to the single mutanT-cells (**Figure 1A**). This result was surprising, as there are additional kinases that can phosphorylate p53Ser18 in an *Atm^−/−^* background. If the decreased proliferation induced by loss of Ser18 is due to regulation by an ATM-independent pathway in MEFs, one could observe that the compound MEFs would have a combined reduced proliferation. We observed a decrease in the proliferation of cells from *Atm^−/−^*; *p53^S18A/S18A^* mice compared to cells from *p53^S18A/S18A^* mice. One possibility is that alternative ATM sites are phosphorylated in the p53^S18A^ protein that contribute to cell proliferation. Alternatively, in the absence of ATM, another kinase can compensate at a secondary site of phosphorylation, but not as efficiently. In addition to growth defects, *Atm^−/−^* mice exhibit somatic growth defects [15]. Interestingly, we observed a significant reduction in the weight of *Atm^−/−^*; *p53^S18A/S18A^* mice compared to *Atm^−/−^* animals at 5–6 months (**Figure 1B**). This is in contrast to our observations that older p53^S18A^ animals are obese [33]. In addition, we observed embryonic lethality in *Atm^−/−^*; *p53^S18A/S18A^* animals (**Table 1**), although not to the same extent seen in *Atm^−/−^*; *p53^−/−^* mice.

Interestingly, the overall survival of *Atm-null* mice was not affected by the presence of a heterozygous or homozygous *p53^S18A^* allele. Of note, the median death of the *Atm^−/−^* mice we observed is longer than reported for some studies [13], [14], [15], but similar to animals maintained in comparable housing conditions [30]. This observation confirms that the conditions of the mouse facility and food can greatly affect the survival of *Atm*-deficient mice.

There was no cooperation in tumorigenesis observed in the compound genotypes compared to *Atm^−/−^* mice (**Figure 2A, B**). The majority of the *p53^S18A^* animals in the *Atm^−/−^* background succumbed to tumors and the tumors the animals developed were similar (**Table 2**). The main tumor developed was thymic lymphoma, which was either lymphoblastic or poorly differentiated in appearance (**Figure 2**). The observation of predominantly lymphoblastic lymphomas in *Atm^−/−^*; *p53^S18A/S18A^* animals is in contrast to the B-cell lymphomas (follicular and centroblastic) observed in *p53^S18A/S18A^* animals [26]. In addition, the thymus was rarely involved in *p53^S18A/S18A^* animals.

The lack of cooperation in tumorigenesis suggests the *Atm^−/−^*; *p53^S18A/S18A^* mice are still able to undergo tumor suppression in B-cells in young animals, which is where *p53^S18A/S18A^* mice develop tumors with a greater latency. It is interesting that the time frame is longer for tumors to arise in *p53^S18A/S18A^* mice than in *Atm^−/−^* or *Atm^−/−^*; *p53^S18A/S18A^* mice, suggesting the mechanism is different. Indeed, tumors in *Atm^−/−^* thymocytes arise from translocations [1], whereas as we linked the tumor suppression by p53Ser18 in B-cells to defective apoptosis [26]. We have also linked apoptosis effects of p53Ser18 to prevention of tumorigenesis by the Myc oncogene [34]. An alternative explanation for the lack of synergy in *Atm-null* and *p53^S18A^* mutant animals may be that the phosphorylation of Ser18 may be redundant in certain cell types and at certain stages, but that later it plays a more critical role. The synergy observed in compound *Atm*-*null* and *p53-null* mice may be due to the fact that there are additional functions of p53 which are lost in *p53-null* animals but still retained in *p53^S18A^* cells. Nonetheless, the rapid demise of the *Atm^−/−^*; *p53^S18A/S18A^* compound animals and the similar tumor type suggests that the loss of *Atm* is dominant over the mutation in p53Ser18.

It has been reported that *Atm^−/+^* mice do not develop spontaneous tumors, whereas *Atm^763del6^* mice have increased cancer risk [35]. In our study, we found that the *Atm^−/+^* mice had a decreased lifespan (**Figure 2B**) and developed tumors (**Table 2**). The tumor penetrance was 23% for *Atm^−/+^* mice and 8% for wild-type mice. Our observation for tumors in *Atm^−/+^* mice is different than that reported, and may be due to a combination of different genetic backgrounds and housing specifications. Different observations have also been reported for studies with radiation-induced tumorigenesis: whereas some studies have reported an increased incidence in radiation-induced tumors in *Atm^−/+^* mice [36], [37], [38], another study has shown no effect of *Atm* heterozygosity on radiation-induced tumors [39]. Our observation is consistent with reports of increased risk of cancer in A-T heterozygous carriers [40].

In addition to a role in tumor suppression, ATM also has a role in motor function. A characteristic of A-T is ataxia. Although *Atm^−/−^* mice do not display overt ataxia, they display defects in motor behavior [15]. In order to assess motor coordination in *p53^S18A^* mice, we examined the performance of the mice on an accelerating rotarod. We used animals at 4–5 months of age (**Figure 3**). All mice used in the study improved rotarod performance over successive trials during the training phase of the experiment as evidenced by an increase in the mean time spent on the rotarod before falling. Thus, mutant and wildtype mice did not significantly differ in motor learning skills (**Figure 3C, D**). Interestingly, during the test trial, female *p53^S18A^* mice showed a significant improved performance on the rotarod (**Figure 3E**). Conversely, male *p53^S18A^* mice exhibited a significant decrease in performance on the rotarod during training (**Figure 3F**). A significant, but modest, increase was seen in the weight of the *p53^S18A^* males, potentially influencing rotarod performance. No weight difference was observed that would affect the results for the female mice. This gender difference suggested the tumor profile could exhibit gender differences. However, we examined the tumor onset and type for the various genotypes and found no gender difference for *Atm* mice on a p53^S18A^ background. Nevertheless, these observations are the first to indicate a gender difference associated with p53 phosphorylation and motor coordination.

In summary, these studies further confirm the essential role of the ATM/p53 pathway in tumor suppression. While loss of p53Ser18 had no increased effects in survival and tumor distribution in *Atm-null* mice, there appears to be a function of p53Ser18 in mediating some of the role of ATM in embryonic survival.
